# Chemical Compositions and Fumigation Effects of Essential Oils Derived from Cardamom, *Elettaria cardamomum* (L.) Maton, and Galangal, *Alpinia galanga* (L.) Willd, against Red Flour Beetle, *Tribolium castaneum* (Herbst) (Coleoptera: Tenebrionidae)

**DOI:** 10.3390/plants13131845

**Published:** 2024-07-04

**Authors:** Ruchuon Wanna, Parinda Khaengkhan, Hakan Bozdoğan

**Affiliations:** 1 Department of Agricultural Technology, Faculty of Technology, Mahasarakham University, Kantarawichai District, Maha Sarakham 44150, Thailand; 2 Resource Management in Agricultural Technology Research Unit, Mahasarakham University, Kantarawichai District, Maha Sarakham 44150, Thailand; 3 Division of Plant Production Technology, Faculty of Agricultural Technology, Kalasin University, Kalasin 46000, Thailand; 4 Vocational School of Technical Sciences, Department of Plant and Animal Production, Kırşehir Ahi Evran University, Kırşehir 40100, Turkey

**Keywords:** Zingiberaceae, essential oil, stored insect pest, plant secondary metabolite, GC-MS

## Abstract

This study explores the use of essential oils from cardamom (*Elettaria cardamomum* (L.) Maton) and galangal (*Alpinia galanga* (L.) Willd) as alternatives to synthetic insecticides for controlling the red flour beetle, *Tribolium castaneum* (Herbst). The chemical compositions of these oils were analyzed using GC-MS, and their fumigation effects were tested in a vapor-phase bioassay. The experiment followed a factorial design with four types of essential oils, namely, those manually extracted from cardamom leaves (MCL) and galangal leaves (MGL) and those commercially produced from cardamom seeds (CCS) and galangal rhizomes (CGR), at seven concentrations (0, 50, 100, 150, 200, 250, and 300 µL/L air). The manually extracted oils yielded 0.6% from cardamom leaves and 0.25% from galangal leaves. MCL contained 28 components, with eucalyptol (25.2%) being the most abundant, while CCS had 34 components, primarily α-terpinyl acetate (46.1%) and eucalyptol (31.2%). MGL included 25 components, mainly caryophyllene (28.7%) and aciphyllene (18.3%), whereas CGR comprised 27 components, with methyl *cis*-cinnamate (47.3%) and safrole (19.8%) as the major constituents. The fumigation bioassay results revealed that CGR was the most effective, demonstrating the highest mortality rates of *T. castaneum* across all the tested periods and concentrations, achieving up to 96% mortality at 168 h with a concentration of 300 µL/L air. Statistical analyses showed significant differences in mortality based on the type and concentration of essential oil, particularly after 96 h. These findings highlight the potential of CGR, with its advantages and differences in chemical composition, as an effective biopesticide against *T. castaneum*, with increasing efficacy over time and at higher concentrations.

## 1. Introduction

Stored-product insects pose a significant threat to global grain stores, causing losses from 10% in temperate regions to nearly 50% in humid tropical areas [1]. Grains and flour, essential carbohydrate sources, are especially susceptible to infestation, leading to diminished quantity and quality. Post-harvest storage, crucial in the agricultural supply chain, is frequently compromised by pest infestations, which exploit favorable conditions in warehouses, resulting in quality degradation and quantitative losses (shrinkage). Insect pests are responsible for 5% to 30% of global agricultural production losses [2]. The red flour beetle is particularly problematic, contaminating grains with shed skins, feces, and dust, affecting cereal flour and stored foods. Overall, post-harvest insect pest losses can reach 30% to 40%, posing a significant threat to both human and livestock health [3]. 

The red flour beetle, *Tribolium castaneum* (Herbst, 1797), is a highly destructive storage pest that significantly impacts stored products and the food economy. Although classified as a secondary pest, it infests damaged grains during harvesting and affects various stored foods, leading to substantial economic losses [4]. Infestations result in agglomeration, discoloration, spoilage [5], and increased temperature and humidity, which promote bacterial and fungal growth. *T. castaneum* is one of the most destructive pests globally due to its broad host range and rapid infestation capabilities. Its presence can trigger allergic responses and product deterioration [6]. Additionally, the beetle has developed resistance to several insecticides, such as malathion and phosphine, complicating control efforts and increasing the risk of food contamination. With its ability to breed year-round in warm climates, *T. castaneum* poses a continuous threat to global food security, especially in tropical and subtropical regions [7]. Effective monitoring and control measures are crucial to mitigate its impact on the cereal value chain and ensure food safety. 

The extensive use of chemical insecticides to protect stored products has led to insect resistance, environmental pollution, and harm to non-target organisms. Although phosphine, malathion, and deltamethrin effectively control pests like *Tribolium castaneum*, their overuse has resulted in resistance, necessitating alternative methods [8,9,10]. The rise in resistance in *T. castaneum* highlights the urgent need for safer pest control options. The reliance on chemical pesticides has also contributed to environmental damage and negative impacts on non-target species. Integrated pest control strategies that combine plant essential oils with chemical pesticides offer a promising solution to reducing environmental harm and promoting sustainable pest management.

Plant secondary metabolites, particularly essential oils, play a vital role in enhancing plant resistance to insects through various mechanisms, such as larvicidal, repellent, and antifeedant effects. These oils are valuable for integrated pest management, as they degrade into non-toxic compounds [8], reducing risks to non-target organisms and humans. Essential oils from aromatic plants effectively control insect pests through fumigation, contact, repellency, and antifeedant actions due to their volatile compounds [11], especially monoterpenes and sesquiterpenes, which act as fast-acting neurotoxins in insects [12]. Several reports indicate that monoterpenoids derived from plants cause insect mortality by inhibiting acetylcholinesterase enzyme (AChE) activity [13,14]. The drawbacks of synthetic insecticides, such as resistance and environmental pollution, have increased interest in botanical insecticides like essential oils, which offer specificity, biodegradability, and low mammalian toxicity. Their natural composition and low persistence also help mitigate the risk of resistance development.

*Elettaria cardamomum* (L.) Maton, commonly known as cardamom, is an aromatic herb from the Zingiberaceae family, renowned as the “Queen of spices” due to its commercial importance. Originally native to India and Sri Lanka, cardamom is now cultivated in various tropical and subtropical regions worldwide [15]. Its distinctive aroma and bioactive compounds lend cardamom significant pharmaceutical and nutraceutical properties [16,17]. It is widely used in food, perfumery, and traditional medicine, serving as a stimulant, stomachic, diuretic, carminative, and anti-infective agent [18]. Cardamom is known for its therapeutic properties, including antispasmodic, stimulant, anthelmintic, antimicrobial, antiviral, antioxidant, anticancer, anti-inflammatory, and insecticidal effects [19,20,21,22]. The essential oil of cardamom, which primarily contains 1,8-cineole and α-terpinyl acetate, has demonstrated toxic effects on various stored-product pests through contact and fumigant actions [23]. 

*Alpinia galanga* (L.) Willd., known as galangal, is an aromatic herb from the Zingiberaceae family, with a history of diverse uses including treating stomachache, antibacterial and antifungal properties, antitumor effects, antiulcer activity, antiallergic effects, antioxidant properties, antiplasmid effects, antimicrobial activities, and insecticidal activity [24,25,26]. Cultivated extensively in Southeast Asia, it is significant in traditional medical systems such as Thai, Ayurveda, Unani, and Chinese medicine [27], and it is valued for its culinary, medicinal, and cosmetic uses. Medically, galangal is known for its anti-inflammatory, antipyretic, emmenagogue, carminative, abortifacient, and aphrodisiac properties [28]. Studies have also explored the insecticidal potential of galangal essential oil against pests like *Bactrocera dorsalis* [26]. 

Therefore, the aim of this study was to assess the chemical compositions and fumigating effects of essential oils from cardamom (*Elettaria cardamomum* (L.) Maton) and galangal (*Alpinia galanga* (L.) Willd) against adult red flour beetles (*Tribolium castaneum* (Herbst)). The chemical compositions were analyzed using gas chromatography–mass spectrometry (GC-MS). Both manually extracted and commercially produced essential oils were studied, providing important advantages and variances to the scope of the study. This comparative analysis of efficiency and chemical composition offers insights into discrepancies in quality, quantity, and potency caused by different extraction methods. Manually extracted essential oils obtained through traditional methods may contain a wider range of compounds. In contrast, commercially produced essential oils provide consistency and purity but may be lack specific constitutes. Manually extracted essential oils have potential advantages in terms of accessibility and cost-effectiveness, making them suitable for small-scale production or local use, especially in regions with limited commercial options. Compositional changes also provide valuable insights into the influence of extraction techniques on insecticidal effectiveness. This holistic approach allows for the assessment of the potential of essential oils as insecticides against *Tribolium castaneum* while accounting for practical considerations such as access and cost. Therefore, this can inform future strategies for pest management.

## 2. Results

### 2.1. Chemical Composition

The yield of the manually extracted *E. cardamomum* essential oil from dried leaves was 0.6% (weight of essential oil/weight of dried leaves × 100). The manually extracted essential oil from cardamom leaves (MCL) contained a total of 28 chemical components, accounting for 90.8%. Table 1 includes a total of 13 compounds, with a combined percentage peak area of 80.3%, indicating that these compounds constituted the majority of the essential oil composition. The primary compounds were eucalyptol (25.2%), *trans*-calamenene (13.4%), and isospathulenol (13.1%). The commercially produced essential oil from cardamom seeds (CCS) comprised a total of 34 constituents, accounting for 98.1%. Table 2 includes a total of seven compounds, with a combined percentage peak area of 92.2%, indicating these compounds made up the majority of the essential oil composition. The main compounds included α-terpinyl acetate (46.1%) and eucalyptol (31.2%). 

In addition to cardamom, eucalyptol (1,8-cineole) is found in other plants such as eucalyptus (*Eucalyptus globulus* Labill.) and rosemary (*Rosmarinus officinalis* L.). Ashokkumar et al. [20] noted that all cardamom accessions contain 1,8-cineole as a significant monoterpene constituent, ranging from 15.2% to 49.4%. Some studies reported lower proportions of α-terpinyl acetate and linalool but higher percentages of 1,8-cineole in cardamom essential oil from various countries, ranging between 25.6% and 26.71% [29,30]. Noshad and Behbahani [31] identified 23 compounds in *E. cardamomum* essential oil, with eucalyptol being the major compound at 31.51%. Alanazi et al. [32] found 19 chemical constituents in *E. cardamomum* essential oil, with monoterpenes like 1,8-cineole (34.3%), α-terpinyl acetate (23.3%), and α-pinene (17.7%) being predominant. The variation in the chemical composition of *E. cardamomum* essential oil can be attributed to factors such as chemotypes, geographical locations, season of plant collection, plant development stage, climate, extraction techniques, plant varieties, and plant part used. These factors can affect the biological activities of the essential oil, with observed fluctuations in volatile constituents like α-terpinyl acetate and 1,8-cineole in cardamom essential oil grown in different zones of the Idukki hills as the season progressed [33,34].

The yield of the manually extracted *A. galanga* essential oil from dried leaves was 0.25% (weight of essential oil/weight of dried leaves × 100). The manually extracted essential oil from galangal leaves (MGL) consisted of 25 components, accounting for 95.7%. Table 3 includes a total of nine compounds, with a combined percentage peak area of 80.4%, indicating these compounds made up the majority of the essential oil composition. The predominant compounds were caryophyllene (28.7%), aciphyllene (18.3%), and α-bisabolene (10.7%). The commercially produced essential oil from galangal rhizomes (CGR) included 27 compositions, accounting for 98.7%. Table 4 includes a total of seven compounds, with a combined percentage peak area of 94.8%, indicating these compounds made up the majority of the essential oil composition. The key compounds were methyl *cis*-cinnamate (47.3%), safrole (19.8%), and *p*-vinylphenylisothiocyanate (11.5%).

The chemical composition analysis of the galangal leaves revealed β-caryophyllene (40.5%) and fenchyl acetate (20.7%) as the main compounds, while cubenol (28.4%), carotol (26.7%), and α-fenchyl acetate (30.5%) were predominant in stem, rhizome, and root [35]. In the Himalayan region of northern India, *A. galanga* exhibited 1,8-cineole (39.4% and 32.5%) and β-pinene (11.9% and 22.7%) as the primary compounds in its rhizome and leaf oils, respectively, with camphor (12.8%) also present in the leaf oil [36,37]. Eucalyptol serves as a marker compound for *Alpinia* spp., with confirmation of its presence in *A. galanga* [38]. Studies also identified 1,8-cineole as a major constituent in the essential oil of *A. calcarata* and *A. galanga* from Tenom, Sabah, Malaysia [39]. Analysis of essential oil from *A. galanga* rhizomes identified 51 components, with eucalyptol and pinenes being prominent [40]. These diverse findings underscore the necessity for further research on plant cultivation and essential oil standardization to address variations influenced by genetic, geographic, physiological, and harvest timing factors within the same plant species [41]. 

By comparing the chemical composition of the essential oils from cardamom, *E. cardamomum*, and galangal, *A. galanga*, it can be seen that the essential oils from MCL and CCS contained eucalyptol as a component and, importantly, they were the same (Table 5). Plant extracts and essential oils containing insecticidal monoterpenoids like limonene, linalool, terpineol, and carvacrol are effective against stored-product insects [42,43]. These natural products disrupt biological pathways and induce tissue injuries, potentially generating free radicals [22]. Monoterpenoids such as α-terpineol, β-pinene, and 1,8-cineol demonstrate fumigant toxicity against various insects like *T. confusum* and *Sitophilus oryzae* [44,45,46]. α-Pinene, p-cymene, α-terpinene, α-terpineol, and terpinene-4-ol also exhibit fumigant toxicity against *S. oryzae* [42]. γ-Terpinene and terpinene-4-ol show promise as fumigants against *T. confusum* and *Ephestia kuehniella* [47]. Additionally, α-pinene and β-caryophyllene possess repellent action against *S. zeamais* [48], while α-pinene demonstrates potential fumigant activity against the same species [49].

Acetylcholinesterase (AChE) is vital in insect and tick nervous systems, breaking down acetylcholine to transmit neuronal signals [50]. Plant-derived volatiles can have neurotoxic effects, potentially inhibiting AChE activity [13]. However, the mode of action of monoterpenoids varies, affecting the octopaminergic nervous system or inhibiting cytochrome P450-dependent mono-oxygenases [51]. Essential oils, like those from *E. cardamomum*, contain terpenoid compounds such as 1,8-cineole, α-pinene, β-pinene, and carvone, which inhibit AChE activity, leading to neuroprotective effects [14]. Additionally, camphene attracts both sexes of *Rhynchophorus ferrugineus* adults, leading to mortality by blocking their air holes and causing asphyxiation [52]. Compounds like linalool, menthol, limonene, and methyl cinnamate exhibit toxicity to insects by inhibiting the AChE enzyme [45,53]. Additionally, 1,8-cineole demonstrates insecticidal properties against post-harvest pests like *T. castaneum* larvae [46]. Other compounds like caryophyllene oxide and methyl cinnamate affect insect respiration [54] and glutathione S-transferase activity, respectively, demonstrating larvicidal activity [55]. It is crucial to note that the activity and mode of action of essential oil compounds may differ from those of individual components.

### 2.2. Effect of Fumigation on Adult Mortality

**Figure 1:**
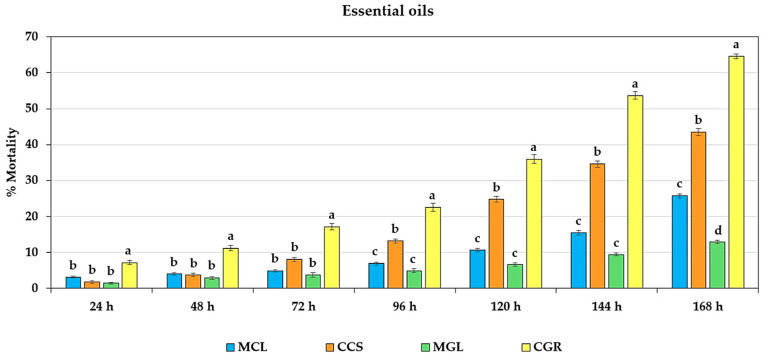
Effect of the essential oil types on mortality of adult *T. castaneum*. Insecticidal activity of MCL (the manually extracted essential oil from cardamom leaves), CCS (the commercially produced essential oil from cardamom seeds), MGL (the manually extracted essential oil from galangal leaves), and CGR (the commercially produced essential oil from galangal rhizomes) against adult *T. castaneum* after exposure within 168 h was found to be significantly different (*p* < 0.01). Means for the same period followed by the same letter were not significantly different (LSD: *p* > 0.05).

The tested essential oil solutions were unable to determine the LC_50_ values for fumigation toxicity against adult *T. castaneum* within 24–72 h. This was possibly due to the low concentration dependence observed at times and no significant differences between concentrations within the same essential oil, which precluded the presentation of the LC_50_ data. However, the types of essential oil had a significantly different (*p* < 0.01) effect on the mortality of adult *T. castaneum* at 24, 48, 72, 96, 120, 144, and 168 h. CGR resulted in the highest mortality of adult *T. castaneum* in all the tested periods, with 7.14 ± 0.52%, 11.14 ± 0.71%, 17.14 ± 0.87%, 22.57 ± 1.13%, 36.00 ± 1.21%, 53.71 ± 1.01%, and 64.57 ± 0.62%, respectively. Significant differences were also observed when compared with MCL, CCS, and MGL (Figure 1).

**Figure 2:**
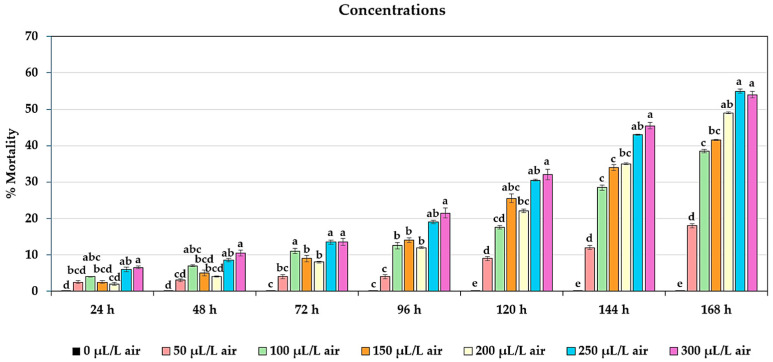
Effect of the concentrations on mortality of adult *T. castaneum*. Insecticidal activity was assessed at seven different concentrations, i.e., 0 µL/L air (control), 50 µL/L air, 100 µL/L air, 150 µL/L air, 200 µL/L air, 250 µL/L air, and 300 µL/L air, against adult *T. castaneum* after exposure within 168 h. Significant difference was found (*p* < 0.05). Means of the same period followed by the same letter were not significantly different (LSD: *p* > 0.05).

The concentrations of the essential oils exhibited a statistically significant difference (*p* < 0.05) in their effect on the mortality of adult *T. castaneum* at 48, 72, 96, 120, 144, and 168 h. A concentration of 300 µL/L air showed the highest mortality of 10.50 ± 0.80%, 13.50 ± 0.94%, 21.50 ± 1.39%, 32.00 ± 1.42%, 45.50 ± 0.82%, and 54.00 ± 0.91%, respectively. However, no statistical difference was found when compared with a concentration of 250 µL/L air. Additionally, a significant difference (*p* < 0.01) was observed at 24 h, with the highest mortality at a concentration of 300 µL/L air being 6.50 ± 0.44%, but no statistical difference was found when compared with a concentration of 250 µL/L air (Figure 2).

**Figure 3:**
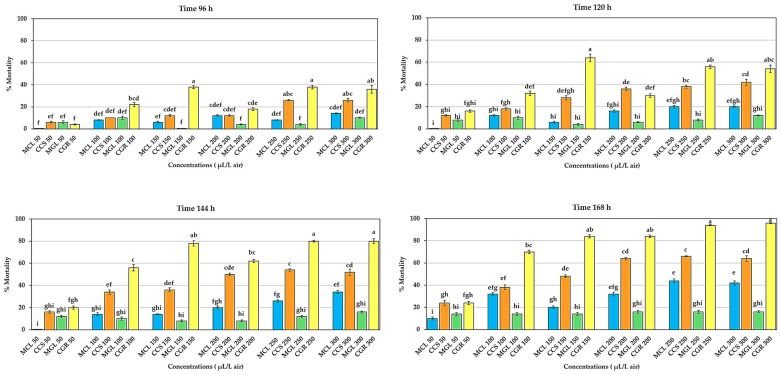
Effect of the interaction between types of essential oils and concentrations on mortality of adult *T. castaneum*. Insecticidal activity was assessed using four types of essential oils at seven different concentrations (with 0 µL/L air as the control, not presented) against adult *T. castaneum* over 24–72 h. Insecticidal activity of MCL (the manually extracted essential oil from cardamom leaves), CCS (the commercially produced essential oil from cardamom seeds), MGL (the manually extracted essential oil from galangal leaves), and CGR (the commercially produced essential oil from galangal rhizomes) against adult *T. castaneum*. Mortality ranged between 2–26%, with no significant differences observed (*p* > 0.05). Significant differences were found within 96–168 h (*p* < 0.05). Means of the same period followed by the same letter were not significantly different (LSD: *p* > 0.05).

The interaction between the types of essential oils and concentrations was found to not affect the mortality of adult *T. castaneum* within 24–72 h after the fumigation bioassay. However, significant differences (*p* < 0.05) were observed in the mortality of adult *T. castaneum* at 96 h due to the interaction between the essential oil types and concentrations. CGR at concentrations of 150 and 250 µL/L air resulted in the highest mortality of 38%, but no difference was observed when compared to a concentration of 300 µL/L air, nor was there a significant difference when compared to CCS at concentrations of 250 and 300 µL/L air. Furthermore, the effect of the interaction between the essential oil types and concentrations on the mortality of adult *T. castaneum* was found to be significantly different (*p* < 0.01) at 120, 144, and 168 h. CGR at a concentration of 150 µL/L air exhibited the highest mortality at 120 h, with 64.00 ± 3.36%, but no difference was observed compared to concentrations of 250 and 300 µL/L air, which resulted in mortalities of 56.00 ± 1.34% and 54.00 ± 3.36%, respectively. At 144 h, CGR at concentrations of 250 and 300 µL/L air had the highest mortality of adult *T. castaneum*, with 80.00 ± 0.71% and 80.00 ± 2.24%, respectively, with no significant difference observed compared to a concentration of 150 µL/L air, where the mortality was 78.00 ± 2.39%. Finally, at 168 h, CGR at a concentration of 300 µL/L air resulted in the highest mortality of adult *T. castaneum*, at 96.00 ± 0.55%. However, no difference was observed compared to concentrations of 150, 200, and 250 µL/L air, which had mortalities of 84.00 ± 1.52%, 84.00 ± 1.14%, and 94.00 ± 0.55%, respectively (Figure 3).

**Figure 4:**
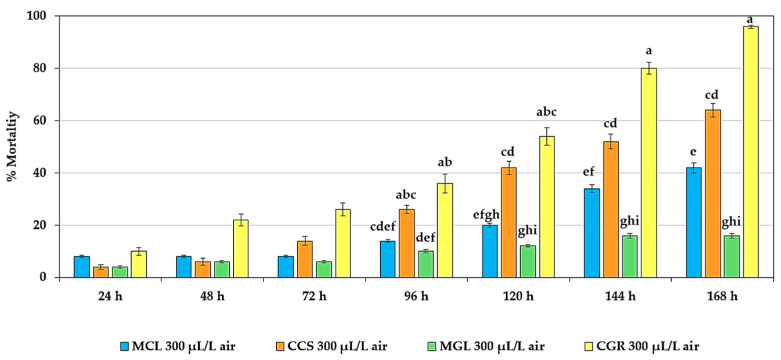
Mortality of adult *T. castaneum* exposed to different essential oils at 300 µL/L air over various time intervals. There were no significant differences observed in the mortality within 24–72 h (*p* > 0.05). Significant differences were found within 96–168 h (*p* < 0.05). Means of the same period followed by the same letter were not significantly different (LSD: *p* > 0.05).

Figure 4 illustrates the mortality of adult *T. castaneum* exposed to different essential oils at a concentration of 300 µL/L air over various time intervals (24, 48, 72, 96, 120, 144, and 168 h). Initially, all the essential oils exhibited low mortality (below 10%). However, the mortality increased steadily over time for all the essential oils, with CGR showing the highest mortality, nearly 90%, within 168 h. Overall, there was a consistent increase in the mortality of all the essential oils, with significant differences becoming more evident after 96 h. CGR at 300 µL/L air emerged as the most effective, especially at 168 h, where it achieved the highest mortality. This trend underscores the effectiveness of essential oils over time, as confirmed by the significant differences in the results.

**Figure 5:**
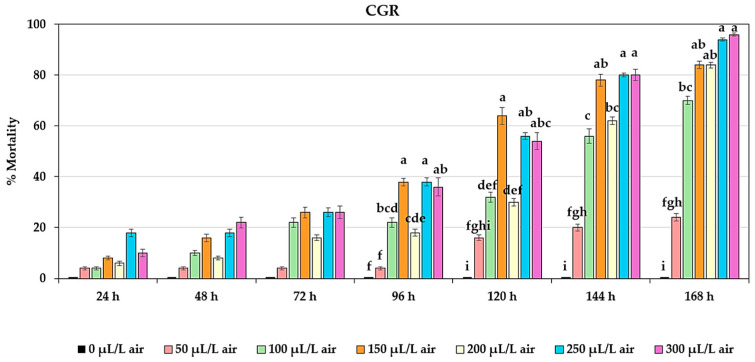
Mortality of adult *T. castaneum* exposed to varying concentrations of CGR (the commercially produced essential oil from galangal rhizomes) over various time intervals. There were no significant differences observed in the mortality (4–26%) within 24–72 h (*p* > 0.05). Significant differences were found within 96–168 h (*p* < 0.05). Means of the same period followed by the same letter were not significantly different (LSD: *p* > 0.05).

Figure 5 depicts the mortality of adult *T. castaneum* exposed to varying concentrations of CGR over different time intervals. At 24 h, the mortality was low across all the concentrations, with the highest concentration being just below 20%. However, as the exposure time increased, the mortality rose for higher concentrations. By 168 h, the highest concentration (300 µL/L air) resulted in the highest mortality, reaching close to 90%. Consistently, higher concentrations led to higher mortality, with the control (0 µL/L air) consistently exhibiting the lowest mortality. This graph illustrates that mortality increased with both CGR concentration and exposure time, with the highest concentration being the most effective, especially at 168 h, Statistical analysis confirmed the significant differences in mortality between the concentrations, particularly after longer exposure times. 

Wang et al. [56] highlight the importance of dosage and exposure duration in the fumigant effect of essential oils. Their study found that the essential oil of CGR, at concentrations of 250–300 µL/L air, resulted in the highest mortality rates (94–96%) of adult *T. castaneum*. These findings are consistent with those of Wu et al. [40], who demonstrated the potent fumigant activity of *A. galanga* rhizome essential oil against *Lasioderma serricorne*, surpassing other essential oils studied previously like those from *Pistacia lentiscus* L., *Elsholtzia stauntonii* Benth., and *Agastache foeniculum* (Pursh) Kuntze in terms of fumigant toxicity against cigarette beetles [57,58]. This heightened efficacy may be attributed to key compounds like methyl cis-cinnamate and safrole found in *A. galanga* rhizomes, known for their repellent, insecticidal, and larvicidal properties [59,60]. 

Specifically, methyl cinnamate has shown insecticidal effects against *S. oryzae* and *Musca domestica* adults, while ethyl cinnamate exhibits antifeedant effects against *Spodoptera littoralis* and *Hylobius abietis*, and propyl cinnamate displayed insecticidal effects on *M. domestica* adults [61,62,63]. Fujiwara et al. [64] highlighted the superior larvicidal activity of methyl cinnamate against *Ae. aegypti*, suggesting the potential of cinnamates as alternatives to conventional insecticides. In addition, the essential oil from galangal rhizome contains safrole as a significant constituent. Huang et al. [65] identified safrole and isosafrole as the major constituents of the essential oils from *Sassafras albidum* (Nutt.) Nees and *Canangium odoratum* (Lam.) Baill. ex King, respectively, which inhibited α-amylase enzyme activity in *T. castaneum* in vitro. Many insects that live on a starch-rich diet depend on the enzymatic activity of α-amylase for survival. Inhibitors of insect α-amylase have already been shown to be effective for controlling insect pests. For example, wheat α-amylase inhibitor causes a significant decrease in larval growth and an increase in mortality in insects [66]. Moreover, *A. galanga* rhizome essential oil contains primary constituents like 1,8-cineol and α-terpineol. Several other studies have identified 1,8-cineole as an effective insecticidal and oviposition repellent across various insect species [67]. Research on essential oils from diverse plants has revealed the deterrent effects of 1,8-cineole on neonate larvae of the codling moth and its efficacy in repelling or controlling adult stages of urban insect pests [55,68].

## 3. Materials and Methods

### 3.1. Insect Rearing

The red flour beetles, *Tribolium castaneum* (Herbst), were sourced from rice bran commonly utilized as animal feed, specifically collected for experimental purposes. Adult males and females were grouped into 30 pairs. They were fed a mixture of coarsely ground wheat flour and rice bran in a ratio of 7:10, housed within 4 L plastic containers, and securely sealed. Ventilation for the containers was scheduled every 3 days. They were maintained within a growth chamber at 26 ± 5 °C and at a relative humidity of 75 ± 5%. The 14-day-old adult red flour beetles were utilized in all bioassays.

### 3.2. Preparation of Essential Oils

The leaves of cardamom (*Elettaria cardamomum* (L.) Maton) and galangal (*Alpinia galanga* (L.) Willd) were collected in November 2022 in Maha Sarakham, Thailand (16°10′38″ N 103°18′03″ E). The plants were identified by their local name, photographed, and collected for the preparation of herbarium specimens, which were deposited at Mahasarakham University (Maha Sarakham, Thailand). Plant identification was carried out primarily based on the taxonomic literature, such as references from the Flora of Thailand. Plant materials were washed, roughly chopped, and dried in a hot-air oven at 65 °C for 2 days, after which they were stored in a plastic bag. Following this, 100 g of the dried plant parts was placed in a 2000 mL flask, and 1000 mL of distilled water was added. The mixture underwent water distillation at 100 ± 20 °C for 4 h using an essential oil extractor. Subsequently, the resulting essential oil underwent purification by centrifuging at 10,000 rpm for 10 min to separate any remaining water from the essential oil. The pure essential oils were then transferred into sealed amber glass vials and stored in a refrigerator at 4 °C until they were ready for further use in bioassays.

The 100% pure natural essential oils extracted from the seeds of cardamom, *E. cardamomum* (CAS-No.85940-32-5), and the rhizome of galangal, *A. galanga* (CAS-No.84625-26-3), were obtained commercially from the Aroma & More Shop, Nonthaburi Province, Thailand. According to the Safety Data Sheets, both products were harmful to aquatic life.

### 3.3. Chemical Compositon Analysis

The essential oils of cardamom and galangal, both manually extracted and commercially produced, were analyzed for their chemical composition using the method outlined by Satongrod et al. [69], employing a Clarus 680 model gas chromatograph–mass spectrometer (PerkinElmer, Waltham, MA, USA). The column utilized was an Rtx-5MS capillary type, with a length of 30 m, diameter of 0.32 mm, and thickness of 1 µm. An essential oil concentration of 100,000 ppm, with a volume of 1 µL, was injected in the split mode (split ratio, 1:100 *v*/*v*). Helium gas served as the carrier gas at a flow rate of 1 mL/minute. The injector temperature was set at 280 °C. For the column conditions, an initial temperature of 45 °C was maintained for 5 min before increasing at a rate of 10 °C/minute to 200 °C, where it was held constant for 5 min. For the mass spectrometry conditions, the electron impact mode was set at 70 eV. Using a quadrupole mass analyzer, the detector temperature was maintained at 250 °C. Spectra were scanned (*m*/*z*) from 40 to 1000 amu. 

The identification of the essential oils was conducted based on their retention indices (RIs), determined concerning a homologous series of C_5_–C_36_ (*n*-alkanes), by comparison of their mass spectra with the reports in the literature using the NIST and Wiley version libraries [70], ensuring a quality match of over 80%. Chemical composition data were analyzed by reading the retention time and % peak area.

### 3.4. Effects of Fumigation Bioassay

Bioassays were performed using the method described by Wongsawas et al. [71]. Fumigation toxicity was assessed through a vapor-phase test conducted in a completely randomized design (CRD) with 5 replicates. Factor A comprised the following 4 types of essential oils: manually extracted essential oil from cardamom leaves (MCL), commercially produced essential oil from cardamom seeds (CCS), manually extracted essential oil from galangal leaves (MGL), and commercially produced essential oil from galangal rhizomes (CGR). Factor B involved the following 7 concentrations: 0 (control, consisting of 100% acetone), 50, 100, 150, 200, 250, and 300 µL/L air, prepared by diluting essential oils with acetone solvent (2, 4, 6, 8, 10, and 12 µL of EO and 48, 46, 44, 42, 40, and 38 µL of 100% acetone for a 40 mL bottle, respectively). The tests were conducted in closed 40 mL glass vials. A total of µL of the test solution was applied onto a 2 cm diameter Whatman No. 1 filter paper disc, which was then allowed to dry at room temperature for 2 min. The filter paper was attached to the bottom surface of the glass vial cap. Five pairs of adult *T. castaneum* were introduced into each test vial, which was tightly sealed with a screw cap. These vials were then stored in a growth chamber at 26 ± 5 °C and at a relative humidity of 75 ± 5%. The number of *T. castaneum* deaths was recorded at 24, 48, 72, 96, 120, 144, and 168 h. Mortality was calculated using the formula [(NC/NT)] × 100, where NC represents the number of dead *T. castaneum* and NT denotes the total number of *T. castaneum* used in the test. If the mortality of *T. castaneum* in the control fell within the range of 5–20%, the mortality of *T. castaneum* in each treatment needed adjustment using Abbott’s formula [72]. Statistical data analysis was conducted using the F-test by analyzing the variance (ANOVA) based on the experimental plan factorial in CRD, and mean comparisons were conducted using the least significant difference method (LSD < 0.05).

## 4. Conclusions

This study demonstrated the insecticidal potential of essential oils from cardamom (*Elettaria cardamomum*) and galangal (*Alpinia galanga*) against *Tribolium castaneum*, with the commercially produced essential oil from galangal rhizomes (CGR) achieving up to 96% mortality at 300 µL/L air after 168 h. These findings highlight CGR as a promising environmentally friendly alternative to synthetic insecticides. However, this study did not experimentally evaluate the mechanisms of action, necessitating future research to confirm the proposed neurotoxic effects and to assess the long-term efficacy, field application, and safety. Addressing these areas will provide a more comprehensive understanding and validation of the use of these essential oils in sustainable pest management.

## Figures and Tables

**Table 1:** The chemical compositions of the manually extracted essential oil from cardamom, *E. cardamomun*, leaves (MCL).

No.	Compound	RI	RT	% Peak Area
1	eucalyptol	1030	8.071	25.2
2	*trans*-*p*-mentha-1(7),8-dien-2-ol	1170	13.841	3.2
3	trans-verbenol	1172	15.560	1.4
4	α-guaiene	1380	26.043	4.1
5	α-cubebene	1400	26.381	3.1
6	*trans*-calamenene	1570	27.587	13.4
7	caryophyllene oxide	1574	29.656	1.4
8	isospathulenol	1575	30.307	13.1
9	ledol	1580	30.747	4.1
10	junenol	1598	30.945	2.7
11	isoaromadendrene epoxide	1621	31.950	1.9
12	epicubenol	1650	32.058	1.6
13	ylangenal	1674	31.783	5.0
	Total			80.3

RI represents retention index, as determined on an Rtx-5MS column. RT represents retention time.

**Table 2:** The chemical compositions of the commercially produced essential oil from cardamom, *E. cardamomun*, seeds (CCS).

No.	Compound	RI	RT	% Peak Area
1	α-pinene	931	6.012	4.7
2	eucalyptol	1030	8.026	31.2
3	linalool	1095	10.291	2.5
4	terpineol	1189	14.046	1.9
5	linalyl acetate	1267	16.388	4.7
6	α-terpinyl acetate	1345	20.907	46.1
7	nerolidol	1560	28.971	1.1
	Total			92.2

RI represents retention index, as determined on an Rtx-5MS column. RT represents retention time.

**Table 3:** The chemical compositions of the manually extracted essential oil from galangal, *A. galanga*, leaves (MGL).

No.	Compound	RI	RT	% Peak Area
1	caryophyllene	1419	24.405	28.7
2	aciphyllene	1425	27.159	18.3
3	α-farnesene	1450	27.415	7.0
4	α-bisabolene	1490	27.822	10.7
5	β-Sesquiphellandrene	1525	28.145	1.9
6	caryophyllene oxide	1582	30.217	4.2
7	isoaromadendrene epoxide	1621	32.814	1.4
8	farnesyl butanoate	1960	39.247	6.1
9	*n*-hexadecanoic acid	2100	44.776	2.3
	Total			80.4

RI represents retention index, as determined on an Rtx-5MS column. RT represents retention time.

**Table 4:** The chemical compositions of the commercially produced essential oil from galangal, *A. galanga*, rhizomes (CGR).

No.	Compound	RI	RT	% Peak Area
1	α-pinene	931	6.200	6.0
2	α-phellandrene	1007	6.973	1.5
3	eucalyptol	1030	7.924	7.7
4	terpineol	1189	14.044	1.1
5	safrole	1209	22.488	19.8
6	*p*-vinylphenylisothiocyanate	1330	22.658	11.5
7	methyl *cis*-cinnamate	1410	23.274	47.3
	Total			94.8

RI represents retention index, as determined on an Rtx-5MS column. RT represents retention time.

**Table 5:** Comparison of the chemical compositions of cardamom, *E. cardamomun*, and galangal, *A. galanga*, essential oils in both the manually extracted and the commercially produced types.

No.	Compound	% Peak Area			
		MCL	CCS	MGL	CGR
1	eucalyptol	25.2	31.2	-	7.7
2	α-terpinyl acetate	-	46.1	-	-
3	methyl *cis*-cinnamate	-	-	-	47.3
4	caryophyllene	-	-	28.7	-
5	safrole	-	-	-	19.8
6	aciphyllene	-	-	18.3	-
7	*trans*-calamenene	13.4	-	-	-
8	isospathulenol	13.1	-	-	-
9	*p*-vinylphenylisothiocyanate	-	-	-	11.5
10	α-bisanolene	-	-	10.7	-
11	caryophyllene oxide	1.4	-	4.2	-
12	terpineol	-	1.9	-	1.1
13	linalool	-	2.5	-	-
14	α-pinene	-	4.7	-	6.0

MCL represents the manually extracted essential oil from cardamom leaves, CCS represents the commercially produced essential oil from cardamom seeds, MGL represents the manually extracted essential oil from galangal leaves, and CGR represents the commercially produced essential oil from galangal rhizomes.

## Data Availability

The original contributions presented in the study are included in the article; further inquiries can be directed to the corresponding author.
